# Ischemia/reperfusion injured intestinal epithelial cells cause cortical neuron death by releasing exosomal microRNAs associated with apoptosis, necroptosis, and pyroptosis

**DOI:** 10.1038/s41598-020-71310-5

**Published:** 2020-09-01

**Authors:** Chien-Chin Hsu, Chien-Cheng Huang, Lan-Hsiang Chien, Mao-Tsun Lin, Ching-Ping Chang, Hung-Jung Lin, Chung-Ching Chio

**Affiliations:** 1 Department of Biotechnology and Food Technology, Southern Taiwan University of Science and Technology, No. 1, Nan-Tai Street, Yungkang District, Tainan City, 710 Taiwan; 2 Department of Emergency Medicine, Chi Mei Medical Center, No. 901, Zhonghua Road, Yongkang District, Tainan City, 710 Taiwan; 3 Department of Senior Services, Southern Taiwan University of Science and Technology, No. 1, Nan-Tai Street, Yungkang District, Tainan City, 710 Taiwan; 4 Department of Environmental and Occupational Health, College of Medicine, National Cheng Kung University, No. 1, University Road, Tainan City, 710 Taiwan; 5 Department of Geriatrics and Gerontology, Chi-Mei Medical Center, No. 901, Zhonghua Road, Yongkang District, Tainan City, 710 Taiwan; 6 Department of Occupational Medicine, Chi-Mei Medical Center, No. 901, Zhonghua Road, Yongkang District, Tainan City, 710 Taiwan; 7 Department of Medical Research, Chi Mei Medical Center, No. 901, Zhonghua Road, Yongkang District, Tainan City, 710 Taiwan; 8 Department of Medicine, Taipei Medical University, No. 250 Wu-Hsing Street, Taipei City, 110 Taiwan; 9 Division of Neurosurgery, Department of Surgery, Chi Mei Medical Center, No. 901, Zhonghua Road, Yongkang District, Tainan City, 710 Taiwan

**Keywords:** Cell death, Gastrointestinal system, Inflammation

## Abstract

To date, there is no good evidence that intestine epithelial cells (IEC) affected by ischemia/reperfusion (I/R) injury are able to cause cortical neuron injury directly. Additionally, it remains unclear whether the neuronal damage caused by I/R injured IEC can be affected by therapeutic hypothermia (TH, 32 °C). To address these questions, we performed an oxygen–glucose deprivation (OGD) affected IEC-6-primary cortical neuron coculture system under normothermia (37 °C) or TH (32 °C) conditions. It was found that OGD caused hyperpermeability in IEC-6 cell monolayers. OGD-preconditioned IEC-6 cells caused cortical neuronal death (e.g., decreased cell viability), synaptotoxicity, and neuronal apoptosis (evidenced by increased caspase-3 expression and the number of TUNEL-positive cells), necroptosis (evidenced by increased receptor-interacting serine/threonine-protein kinase-1 [RIPK1], RIPK3 and mixed lineage kinase domain-like pseudokinase [MLKL] expression), and pyroptosis (evidenced by an increase in caspase-1, gasdermin D [GSDMD], IL-1β, IL-18, the apoptosis-associated speck-like protein containing a caspase recruitment domain [ASC], and nucleotide oligomerization domain [NOD]-like receptor [NLRP]-1 expression). TH did not affect the intestinal epithelial hyperpermeability but did attenuate OGD-induced neuronal death and synaptotoxicity. We also performed quantitative real-time PCR to quantify the genes encoding 84 exosomal microRNAs in the medium of the control-IEC-6, the control-neuron, the OGD-IEC-6 at 37 °C, the OGD-IEC-6 at 32 °C, the neuron cocultured with OGD-IEC-6 at 37 °C, and the neurons cocultured with OGD-IEC-6 at 32 °C. We found that the control IEC-6 cell s or cortical neurons are able to secrete a basal level of exosomal miRNAs in their medium. OGD significantly up-regulated the basal level of each parameter for IEC-6 cells. As compared to those of the OGD-IEC-6 cells or the control neurons, the OGD-IEC-6 cocultured neurons had significantly higher levels of 19 exosomal miRNAs related to apoptosis, necroptosis, and/or pyroptosis events. Our results identify that I/R injured intestinal epithelium cells can induce cortical neuron death via releasing paracrine mediators such as exosomal miRNAs associated with apoptosis, necroptosis, and/or pyroptosis, which can be counteracted by TH.

## Introduction

It is generally believed that bacterial translocation and gut-origin sepsis cause systemic infections complications and multiple organ deficiency syndromes (MODS) in surgical and critically ill subjects^1^. The gut microbiota plays a role in both normal central nervous system (CNS) development and pathogenesis of anxiety, memory loss^2^, and other stress-related psychiatric disorder^3^. Intestinal ischemia/reperfusion (I/R) caused by clamping the superior mesenteric artery^4^ or by resuscitation following hemorrhagic shock (HS)^5^ induces cerebral injury and memory dysfunction in rats^4^. To date, there is no good evidence that the intestine epithelial cells affected by I/R injury are able to cause neuronal damage directly. Additionally, it remains unclear whether the brain injury caused by I/R injured intestine epithelial cells, like rat heart, can be affected by therapeutic hypothermia (TH)^6^.

To address these questions, we cocultured the oxygen–glucose deprivation (OGD)-infected intestinal epithelial cell lines (IEC-6) with rat primary cortical neuronal cells in an in vitro coculture system under normothermia (37 °C) or TH (32 °C) conditions. Exclusive use of the transwell coculture system eliminates the possibility of determining which cells are responsible for miRNA release and other events. Intestinal permeability, cortical neuronal death and synaptotoxicity, and the expression of cortical apoptosis-related proteins (e.g., caspase-3 and caspase-8)^7^, necroptosis-related proteins (e.g. receptor-interacting protein kinase-1 [RIPK1], RIPK3 and mixed lineage kinase domain-like protein [MLKL])^7^ and pyroptosis-related proteins (e.g., caspase-1, gasdermin D [GSDMD], apoptosis-associated speck-like protein containing a caspase recruitment domain [ASC] and a nucleotide oligomerization domain [NOD]-like receptor protein [NLRP-1], interleukin-1β [IL-1β], and interleukin-18 [IL-18])^8^ were measured. We also performed quantitative real-time reverse transcription PCR (qRT-PCR) to quantify the genes encoding 84 exosomal microRNAs (miRNAs)^9^ in the cocultured medium of the control IEC-6, the control neurons, the OGD-IEC-6 at 37 °C, the OGD-IEC-6 at 32 °C, the neurons cocultured with OGD-IEC-6 at 37 °C, and the neurons cocultured with OGD-IEC-6 at 32 °C.

## Materials and methods

**Figure 1:**
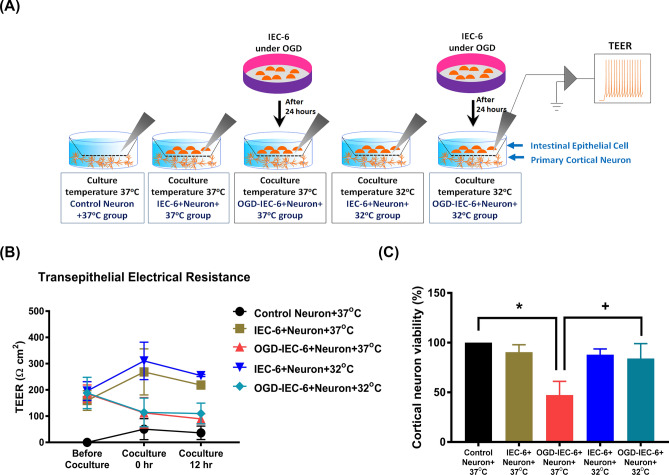
(**A**) and (**B**)The epithelial permeability of DMEM at 37 °C, IEC-6 cells at 37 °C, OGD-preconditioned IEC-6 cells at 37 °C, IEC-6 cells at 32 °C, and OGD-preconditioned IEC-6 cells at 32 °C was assayed by determining transepithelial electrical resistance (TEER). (C) The viability of cortical neurons treated with medium alone at 37 °C was defined as 100%. Measurements were made in triplicate, and each bar represents the mean ± S.D. *P < 0.01, OGD-preconditioned IEC-6 cells+neurons at 37 °C vs. controls; ^+^*P* < 0.05, OGD-preconditioned IEC-6 cells+neurons at 32 °C.

All authors had access to the study data and reviewed and approved the final manuscript. All in vitro studies are from at least 3 replicate experiments. Schematic diagrams showing the in vitro experimental designs are shown in Fig. 1. All the antibodies and commercial kits used in this study are summarized in Supplementary Table S1.

### Primary cortical neuron cultures

Primary cultured cortical neurons were obtained from embryonic (E18D) Sprague–Dawley rat fetuses and maintained for eight to ten days before being used for experiments as detailed previously^10^. Briefly, samples of the cerebral cortex were dissected and digested with EDTA, then dissociated with a Pasteur pipette. The dissociated cells were resuspended in neurobasal medium (Gibco, Thermo Fisher Scientific Inc., Waltham, MA, USA) containing 2% B27 (Gibco), 0.5 mM glutamine and 50 U/ml penicillin/streptomycin and plated onto poly-D-lysine-coated culture dishes. The media was first refreshed 24 h after plating and half-replaced with fresh media every 3 days. Cultured cortical neurons were kept for 8–10 days at 37 °C in a 5% CO_2_ incubator before being used for experiments.

### IEC-6 cell cultures

Cells from the rat intestinal epithelial cell line No. 6 (IEC-6) at the 13^th^ culture passage were obtained from the American Type Culture Collection (ATCC, Manassas, VA, USA) and maintained in Dulbecco’s modified Eagle’s medium (Gibco). Cells were cultured at 37 °C in a humidified atmosphere with 5% CO_2_. All experiments used cells at the 17th to 19th passage and were performed in at least triplicate. The condition of the cells was observed using phase-contrast microscopy, and healthy cells were sub-cultured for subsequent experimentation.

### Oxygen–glucose deprivation (OGD) and the epithelial-neuron coculture model

IEC-6 cells were washed with phosphate-buffered saline (PBS), switched to Roswell Park Memorial Institute (RPMI) medium without glucose and serum, and placed in a 0.2% O_2_ hypoxia chamber for 24 h at 37 °C to induce OGD. Twenty-four hours after completing the OGD induction protocol, IEC-6 cells on transwell insert (with 0.4-μm-pore polyethylene terephthalate membrane; #353493, Falcon; BD Biosciences, Franklin Lakes, NJ, USA) were cocultured with primary cortical neurons as shown in Fig. 1A. More specifically, one porous filter containing IEC-6 cells cultured at confluence was placed above cortical neurons cultured in a 12-well plate, and cells were then cocultured for 12 h. IEC-6 cells in the control group were not subjected to OGD. To explore the effects of hypothermia on epithelial-neural interactions following OGD, cortical neurons alone at 37 °C, cortical neurons plus untreated IEC-6 cells at 37 °C, cortical neurons plus OGD-treated IEC-6 cells at 37 °C, and cortical neurons plus OGD-treated IEC-6 cells at 32 °C were cultured for 12 h. After coculturing, cells and medium were collected for cell viability, apoptosis, necroptosis, pyroptosis, cytokines, and exosome microRNA assays.

### Cell viability assay

Cell viability after coculture was determined using the 3-(4,5-dimethylthiazol-2-yl)-2,5-diphenyltetrazolium bromide (MTT; Sigma-Aldrich) assay^11^. After coculture, neurons were treated with MTT solution (50 μl/well of 5 mg/ml MTT solution) for 2 h at 37 °C. The dark blue formazan crystals formed in intact cells were solubilized with lysis buffer. The absorbance of the sample was read at 570–630 nm with a spectrophotometer (Molecular Devices, Sunnyvale, CA, USA). The A570/A630 ratio, which is directly related to the number of viable cells in each well, was calculated. The results are expressed as the percent (%) of MTT reduction, assuming that the absorbance of control cells at 37 °C was 100%.

### Transepithelial electrical resistance (TEER) assay

Intestinal epithelial cell permeability was assayed by determining TEER using a Millipore electric resistance system (ERS-2; Merk Millipore, Billerica, MA, USA) and calculated as Ω/cm^2^ according to the method detailed previously^3^.

### Immunofluorescence analysis of synaptophysin-containing neurons

Cortical neurons were seeded on sterile poly-l-lysine coverslips placed in 6-well culture plates, incubated with mouse microtubule-associated protein 2 (MAP-2; a neuronal marker; Santa Cruz) and rabbit synaptophysin antibodies (Santa Cruz) and diluted in 5% BSA overnight at 4 °C. Then, cortical neurons were incubated with the appropriate secondary antibodies (Alexa Fluor 568-conjugated goat anti-mouse IgG or Alexa Fluor 488-conjugated goat anti-mouse) for 1 h at room temperature. The neurons were also stained with 4′,6-diamidino-2 phenylindole dihydrochloride (DAPI, Sigma-Aldrich) as a nuclear marker. Digital images were captured with a 20 × objective (N.A. 0.75) by a fluorescence microscope system (Carl Zeiss Microscopy GmbH, Jena, Germany) with Zen Software (Carl Zeiss). The experiments were repeated three times. Synaptophysin and MAP-2 positive cells were counted, and the fluorescence intensity was quantified using ImageJ v.51j8 software (https://rsbweb.nih.gov/ij/). For the cell count, data are presented as the percentage of the total synaptophysin-containing neurons number in four fields. For the fluorescence intensity quantification, all images were conversed with grayscale and then analyzed by density measurement. A fixed threshold rage of 10–150 was chosen to highlight the fluorescence staining signals. Data are normalized to the average fluorescence intensity of the control neuron^12^.

### Terminal deoxynucleotidyl transferase (TdT)-mediated dUTP nick end-labeling (TUNEL) assay

In vitro labeling of fragmented DNA in cultured cortical neurons was performed using the ApoAlert DNA Fragmentation Assay Kit (Clontech Laboratories Inc., CA, USA) according to the manufacturer's instructions. This assay allows the direct labeling of the fragmented DNA with fluorescein. Twelve hours with or without IEC-6 or OGD-IEC-6 coculture, the neurons were fixed and incubated with a terminal deoxynucleotidyl transferase (TdT) reaction buffer at 37 °C. Briefly, blunt ends of double‐stranded DNA molecules were enzymatically labeled with fluorescein‐dUTP, and all the neurons were stained with propidium iodide at a concentration of 750 ng/ml. DNase-treated (RNase-Free DNase Set, # 79254, Qiagen) neuron was used as a positive control. Neurons were not treated with TdT as a negative control. All cell nuclei were counterstained with DAPI. We determined the level of apoptotic neurons by counting MAP-2 with TUNEL-positive cells using a fluorescence microscope (Carl Zeiss) equipped with fluorescein isothiocyanate filters, in accordance with the manufacturer's recommendations. The experiments were repeated three times. Data are presented as the percentage of the total apoptotic neuron number in four fields. The fluorescence intensity of TUNEL in MAP-2-positive cells was also quantified using ImageJ v.51j8 software (https://rsbweb.nih.gov/ij/). All images were conversed with grayscale and then analyzed by density measurement, as described in the section mentioned above.

### Immunoblot analysis of apoptotic, necroptotic, and pyroptotic protein levels in cortical neurons

The total protein concentrations of the cortical neurons treated with lysis buffer (Cell Signaling Technology, Danvers, MA) were determined using a bovine serum standard curve as a reference. A total of 40 μg of protein was evaluated via 10% SDS-PAGE and electrotransferred onto a nitrocellulose membrane. Membranes were blocked overnight at 4 °C and then incubated in a primary antibody in its respective blocking solution for 2 h at room temperature. The primary antibodies used were anti-caspase-3, anti-caspase-8, anti-RIPK1, anti-RIPK3, anti-pMLKL, anti-caspase-1, anti-NLRP-1, anti-NLRP-3, anti-ASC, anti-IL-1β, anti-GSDMD, anti- zonula occludens 1 (ZO-1), anti-claudin-1, and anti-β-actin. Membranes were incubated with a secondary antibody diluted 1:5,000 in blocking buffer. The density of Western blot bands was quantified using an image analysis system (Image Pro-Plus; Media Cybernetics, USA). Each protein levels were determined after normalizing with β-actin.

### Enzyme-linked immunosorbent assay (ELISA)

The concentrations of tumor necrosis factor-α (TNF-α), interleukin-6 (IL-6), and interleukin-18 (IL-18) in the media or cortical neurons were determined by commercialized ELISA kits (R&D Systems, Minneapolis, MN, USA) according to the manufacturer’s instructions.

### Exosomes isolation and microRNA analysis

Total exosomes were isolated from either conditioned neuron culture medium or conditioned IEC-6 culture medium. The total cell culture medium was harvested from 8 × 10^7^ cells of each group and centrifuged at 2000×*g* for 30 min to remove suspended cells and debris. Total exosome isolation (from cell culture media) reagent (Invitrogen) was used to isolate total exosomes following the manufacturer’s instructions. Total RNA was isolated from exosome using a miRNeasy Mini kits (Qiagen, Valencia, CA, USA) and finally eluted into 30 μl of heated elution solution. Purified exosomes from neurons or IEC-6 cells were identified using a cluster of differentiation (CD)9 (#STJ92147, St. John Laboratory Ltd., London, UK) by Western blot analysis (Supplementary Figure S1). The purity and concentration of all exosomal RNA samples were evaluated by the absorbance ratio at 260/280 nm, determined using a spectrophotometer (Molecular Devices, Sunnyvale, CA, USA). Exosomal miRNA expression profiles were analyzed using the Rat Inflammatory Response & Autoimmunity miScript miRNA PCR Array platform (Qiagen) which contain miScript primers for 84 of well-characterized miRNAs and 2 synthetic spike-in control miRNA as it has no mammalian homologue (cel-miR-39-3p), 6 internal controls miRNAs (SNORD61, SNORD68, SNORD72, SNORD95, SNORD96A, RNU6-6P), 2 miRNA reverse transcription control (miRTC), and 2 Positive PCR control (PPC). qRT-PCR of miRNA was performed using a QuantStudio 3 Real-Time PCR System (Applied Biosystems, Waltham, MA, USA). The relative expression of each miRNA was calculated using the comparative cycle threshold method (2^-△△CT^) and is presented in fold change relative to the Control-IEC-6 group. The putative genes were subjected to functional and pathway enrichment analysis and the potential regulatory relationships between miRNAs and target genes using the Kyoto Encyclopedia of Genes and Genomes (KEGG). The threshold of significance was defined as *P* < 0.05 for KEGG analyses.

### Statistics

Data are presented as the mean ± S.E.M. Data with non-normal distribution were analysed by the Kruskal–Wallis test with Dunn’s post-hoc test. We used GraphPad Prism (version 7.01 for Windows; GraphPad Software, San Diego, CA, USA) to analyze the data and set the statistically significant level at *P* < 0.05.

### Ethical approval

Primary cortical neuron cultures were prepared from pregnant Sprague–Dawley rats from (BioLASCO Taiwan Co., Ltd.) at embryonic day 18 (E18D). All animal experiments were conducted under protocols approved by the Institutional Animal Care and Use Committees of Chi Mei Medical Center, Tainan City, Taiwan (approved IACUC no. 106121110) in accordance with the National Institutes of Health Guide for the Care and Use of Laboratory Animals. We used the ARRIVE checklist when writing our report.

## Results

### OGD-preconditioned IEC-6 cell-induced cell death in cortical neurons can be prevented by cooling therapy

We performed an in vitro study to ascertain whether the mediators released from IEC-6 cells preconditioned with OGD cause primary cortical neuron death under therapeutic hypothermia or normothermia conditions. Figure 1A shows the schematic representation of the five in vitro coculture system conditions used in this study. In this system, the IEC-6 cells on a porous filter were placed above cultured primary cortical neurons. The two different types of cells were not in direct contact with each other, but rather any effects of one cell type on the other occurred as a result of the secretion of soluble factors. Therefore, any cross-talk between the two cell types must be via soluble factors secreted from each cell line.

As shown in Fig. 1B, IEC-6 cell monolayers preconditioned with OGD for 24 h were cocultured with cortical neurons at 37 °C. It was found the TEER value (an indicator for epithelial permeability) of OGD-IEC-6+Neuron+37 °C group was significantly lower than that of untreated control IEC-6 cells cocultured with neurons at 37 °C (IEC-6+Neuron+37 °C group). The tight junction-associated proteins (ZO-1 and Claudin-1) expression were also decreased after OGD treatment in IEC-6 (Supplemental Fig. 3S). Moreover, the viability of cortical neurons cocultured with OGD-preconditioned IEC-6 cells at 37 °C (OGD-IEC-6+Neuron+37 °C group) was significantly lower than that of cortical neurons cocultured with non-OGD preconditioned control IEC-6 cells (IEC-6+Neuron+37 °C group) (Fig. 1C). Although therapeutic hypothermia did not affect the OGD-induced decrease in TEER and tight junction-associated proteins (ZO-1 and claudin-1) expression in IEC-6 cells (Fig. 1B and Supplemental Fig. 3S), it significantly improved the viability of cortical neurons cocultured with IEC-6 cells preconditioned with OGD (Fig. 1C).

### Therapeutic hypothermia attenuates OGD-preconditioned IEC-6 cell-induced synaptotoxicity and apoptosis in cultured primary cortical neurons

**Figure 2:**
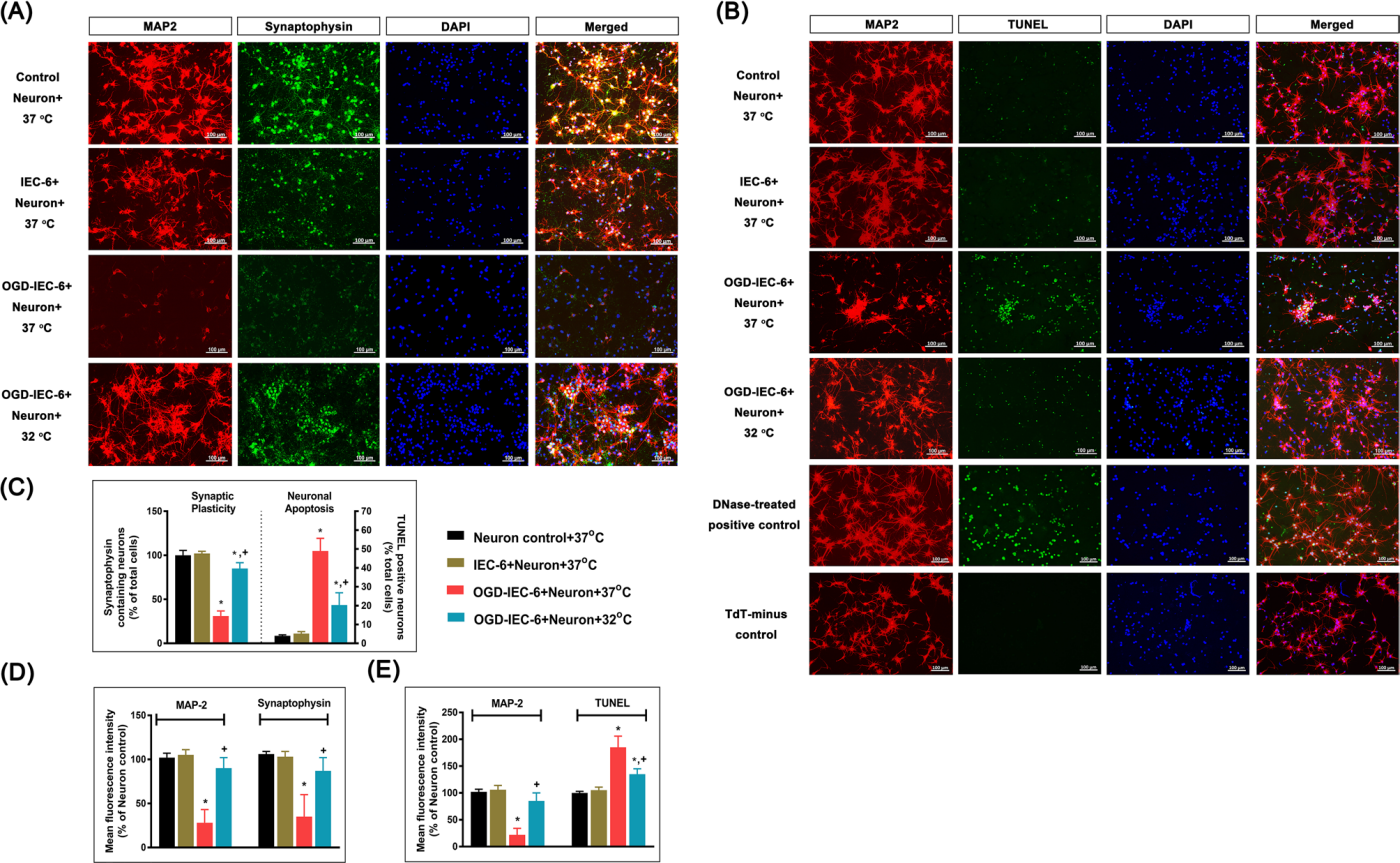
(**A**) Representative immunofluorescence images showing colocalization of MAP2 (neuron-specific nuclear protein marker, red) and synaptophysin (synaptic vesicle protein marker, green) in cortical cells in each experimental group. (**B**) Representative immunofluorescence photomicrographs of MAP2 (red) and TUNEL (apoptosis marker, green) positive cells in each experimental group. The DNase-treated neuron was used as a positive control. Neurons were not treated with TdT as a negative control. All cell nuclei were counterstained with DAPI (blue). (**C**) Quantification of synaptophysin in neurons were evaluated by the ratio of MAP2 and synaptophysin double-positive cells (yellow) to DAPI positive cells (blue). Percentages of apoptotic neurons were evaluated by the ratio of TUNEL and MAP2 double-positive cells (yellow) to DAPI positive cells (blue). Quantification of (**D**) MAP2 and synaptophysin staining and (E) MAP2 and TUNEL staining fluorescence intensity from each group were normalized to an average intensity of the Neuron control+37 °C group. Each bar represents the means ± S.D. of 6 independent cultures in each experimental condition. *p < 0.05, OGD-IEC-6+Neuron+37 °C vs. Control Neuron+37 °C or IEC-6+Neuron+37 °C; ^+^*p* < 0.05, OGD-IEC-6+Neuron+37 °C. Scale bar, 100 μm.

In this study, we aim to ascertain whether therapeutic hypothermia attenuates OGD-preconditioned IEC-6 cells-induced cortical neuronal injury in the coculture system. We observed both synaptotoxicity (as evidence by decreased expression of the dendritic marker MAP2 and of the synaptic protein synaptophysins), and neuron apoptosis (as evidenced by accumulation of MAP2- and TUNEL-positive cells) in primary cultures of cortical neurons co-cultured with OGD-preconditioned IEC-6 cells (Fig. 2A, B). Indeed, Fig. 2C, D shows that the number of synaptophysin-containing dendrites of cortical neurons and MAP2 and synaptophysin fluorescence intensity in OGD-IEC-6+Neuron+37 °C group was significantly lower than that of IEC-6+Neuron+37 °C group. Similarly, we observed an increase in apoptotic neurons and TUNEL fluorescence intensity cocultured with OGD-preconditioned IEC-6 cells relative to non-OGD-preconditioned counterparts (Fig. 2C, E). However, therapeutic hypothermia significantly reversed both the synaptotoxicity and apoptosis in cortical neurons cocultured with OGD-preconditioned IEC-6 cells (Fig. 2).

### Therapeutic hypothermia attenuates the increased expression of apoptosis-related, necroptosis-related, and pyroptosis-related proteins in cortical neurons cocultured with OGD-preconditioned IEC-6 cells

**Figure 3:**
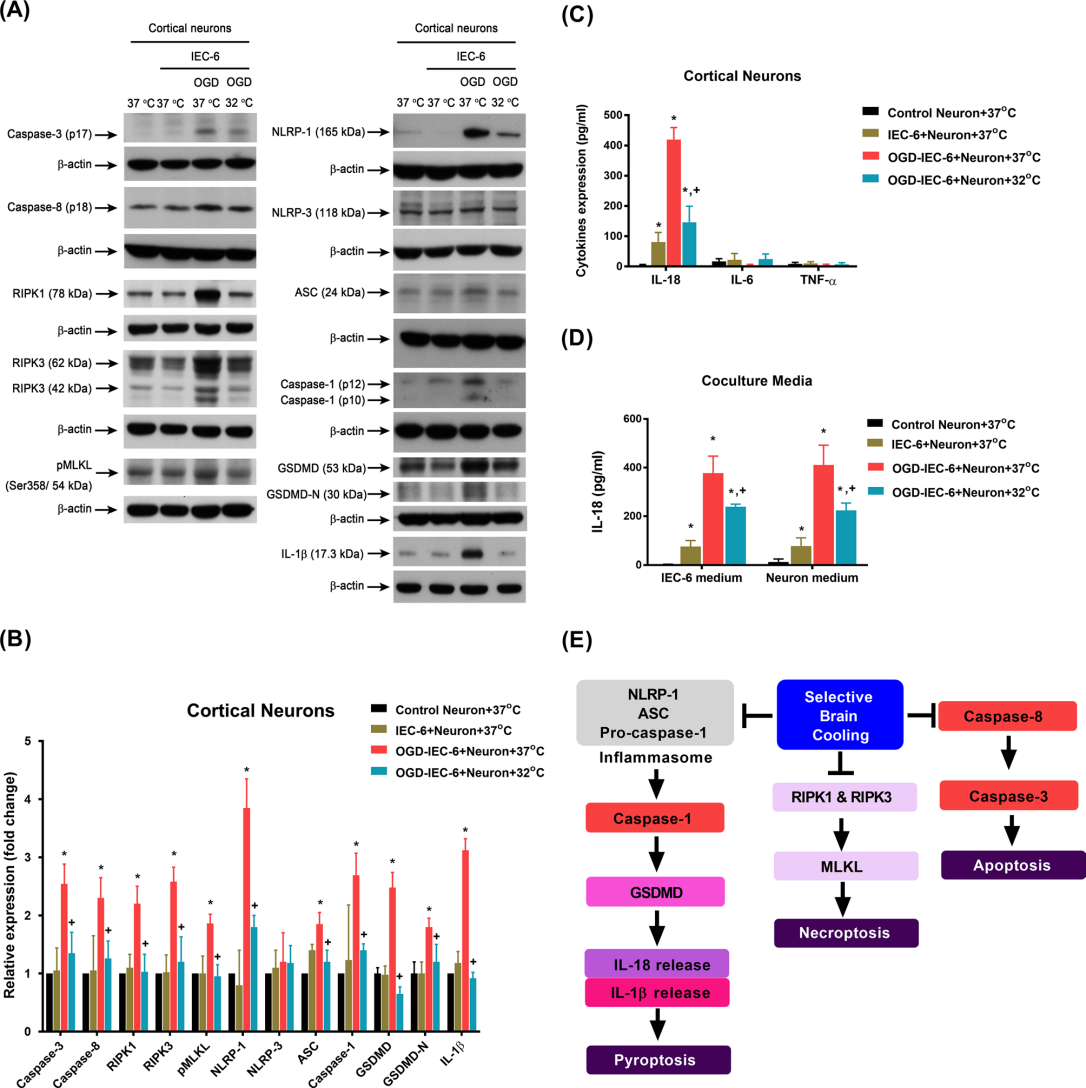
(**A**) Representative Western blots showing the expression levels of various parameters across experimental groups. β-actin was used as a loading control. (**B**) A representative immunoblot is shown, and bands of various parameters were quantified by densitometry and normalized to β-actin. (**C**) Bar graph quantifying the expression levels of cytokines in cortical neurons across experimental groups. (**D**) Bar graph quantifying the expression levels of extracellular IL-18 in co-culture media obtained from IEC-6 cells and primary cortical neurons. All measurements were made in triplicate, and each bar represents the mean ± S.D. *p < 0.05, compared with the Control Neuron+37 °C group; ^+^*p* < 0.05, compared with the OGD-IEC-6+Neurons+37 °C group. (**E**) We hypothesize that the NLRP-1 inflammasome leads to caspase-1 activation and subsequent upregulation of proinflammatory cytokines such as IL-1β and IL-18, triggering pyroptosis, which ultimately induces HS pathology through several downstream effects in the brain. At the same time, apoptosis and necroptosis can also be observed. Our present data suggest that caspase-3-induced apoptosis, MLKL-induced necroptosis, and GSDMD-induced neuronal pyroptosis may provide a molecular basis for brain-gut interactions during HS. Hypothermia therapy may protect against HS by suppressing both the apoptosis, necroptosis and pyroptosis pathways.

Twenty-four hours after OGD, IEC-6 cells were cocultured with primary cortical neurons for 12 h. After coculturing, cells and medium were collected for analyses of apoptosis (evidenced by caspase-3, caspase-8, and TUNEL positive cells), necroptosis (evidenced by RIPK-1, RIPK3, and pMLKL) and pyroptosis (evidenced by NLRP-1, ASC, caspase-1, IL-18, IL-1β, GSDMD and N-terminal fragment of GSDMD [GSDMD-N]) (Fig. 3A, for original images of the blots please see Supplementary Figure S2). Figure 3B shows that the expression of apoptosis-related, necroptosis-related, and pyroptosis-related proteins in the cortical neurons cocultured with the OGD-preconditioned IEC-6 cells at 37 °C (OGD-IEC-6+Neuron+37 °C group) were significantly higher than those from the cortical neurons cocultured with the non-OGD-preconditioned controls (IEC-6+Neuron+37 °C group) (Fig. 3A, B). Therapeutic hypothermia (OGD-IEC-6+Neuron+32 °C group) significantly reduced the overexpressions of these proteins in cortical neurons cocultured with OGD-preconditioned IEC-6 cells groups (OGD-IEC-6+Neuron+37 °C group) (Fig. 3A, B). Additionally, ELISA assay revealed that the expression of IL-6 and TNF-α in cortical neurons was similar across all experimental groups (Fig. 3C). However, IL-18 expression in both the cortical neurons and conditioned media was significantly higher in the OGD-IEC-6+Neuron+37 °C than those of the IEC-6+Neuron+37 °C (Fig. 3D). Compared to those of the OGD-IEC-6+Neuron+37 °C group, the levels of IL-18 in both the neurons and culture media were significantly lower in OGD-IEC-6+Neuron+32 °C group (Fig. 3D). Figure 3E summarizes that therapeutic hypothermia significantly inhibits both cortical neuronal apoptosis, necroptosis and pyroptosis induced by intestinal ischemia and reperfusion.

### Therapeutic hypothermia reduces the overexpression of apoptosis, necroptosis, and pyroptosis related exosomal miRNAs from the cocultured medium of OGD-preconditioned IEC-6 cells and neurons

**Figure 4:**
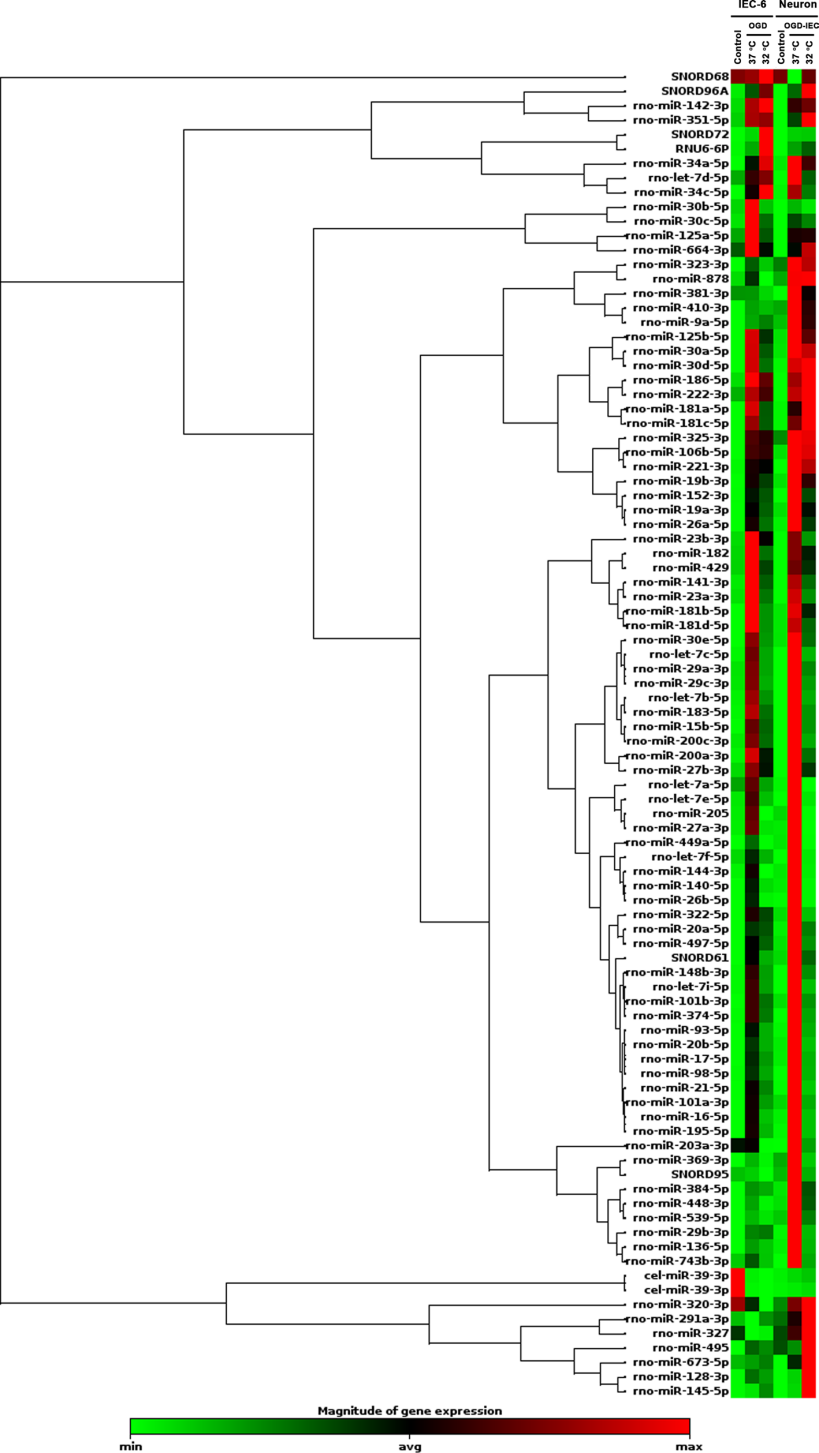
Clustering heat map of the 84 exosomal miRNA expression profiles in the cultured medium by qRT-PCR-based array analysis obtained from Control-IEC-6 cells, OGD preconditioned-IEC-6 at 37 °C (OGD-IEC-6+37 °C), OGD preconditioned-IEC-6 at 32 °C (OGD-IEC-6+32 °C), control cortical neurons at 37 °C (Control-Neuron+37 °C), cortical neurons cocultured with OGD-preconditioned IEC-6 cells at 37 °C (OGD-IEC-6+Neuron+37 °C) and cortical neurons cocultured with OGD-preconditioned IEC-6 cells at 32 °C (OGD-IEC-6+Neuron+32 °C). Relative miRNA expression is given according to the color scale shown at the bottom: red indicates a higher expression level than average, and green indicates a lower expression level than average.

We used a qRT-PCR-based array to analyze 84 miRNA expression levels in the medium of non-OGD IEC-6 cultured alone (Control-IEC-6 group), OGD preconditioned-IEC-6 at 37 °C (OGD-IEC-6+37 °C), OGD preconditioned-IEC-6 at 32 °C (OGD-IEC-6+32 °C), control cortical neurons at 37 °C (Control Neuron+37 °C), cortical neurons cocultured with OGD-preconditioned IEC-6 cells at 37 °C (OGD-IEC-6+Neuron+37 °C) and cortical neurons cocultured with OGD-preconditioned IEC-6 cells at 32 °C (OGD-IEC-6+Neuron+32 °C) (Fig. 4). The fold increase for each parameter for the Control-IEC-6 group is “1”. Compared to the Control-IEC-6 group, the medium of the OGD-IEC-6+37 °C group had 78 up-regulated miRNAs, 2 down-regulated miRNAs and 4 unaltered miRNA (Supplementary Table S2). In comparison with those of the OGD-IEC-6+37 °C group or Control Neuron++37 °C group, the medium of the OGD-IEC-6+Neuron+37 °C group had significantly 19 upregulated (2 or more fold increase) miRNAs (Table 1). The increased levels of these 19 upregulated miRNAs in the medium of the OGD-IEC-6+37 °C and the OGD-IEC-6+Neuron+37 °C were all significantly reduced by TH (32 °C) therapy (Table 1).

**Table 1:** The 19 miRNAs significantly upregulated (two-fold or more) in cortical neurons 12 h coculturing with OGD-IEC-6 cells (determined by qRT-PCR).

microRNA	KEGG pathway target gene	Fold changes [compared to (Control-IEC-6) group]					Gene function (reference)
		OGD-IEC-6+37 °C	OGD-IEC-6+32 °C	Control-Neuron+37 °C	OGD-IEC-6+Neuron+37 °C	OGD-IEC-6+Neuron+32 °C	
rno-miR-384-5p	Ppp3cb Pik3r2 Casp3 Prkar2b Casp8 Tp53 Il1a Pik3cd Ppp3r1	164.6*	125.4	35.6	783.0^†^	267.7^‡^	Enhances ER stress-induced cell apoptosis, and upregulates cleaved caspase-3 expression^16^
rno-miR-136-5p	Ppp3r2 Ppp3r1 Fadd Chuk	119.9*	67.7	18.1	629.5^†^	88.0^‡^	Modulates the inflammatory and immune responses and downregulates Bcl-2^17^
rno-miR-9a-5p	Bcl2 Ikbkb Prkar2b CFLAR Cyct Dffa Pik3cd Nfkb1 Irak1 Capn1	67.4*	100.2	49.6	403.9^†^	239.7^‡^	Increases the levels of pro-apoptotic regulator Fas ligand (FasL)^19^
rno-miR-369-3p	Tnf Bcl2 Casp3 Prkx Il1a Ngf Casp6 Ppp3r1	35.8*	5.7	40.6	248.0^†^	25.3^‡^	Induces apoptosis as well as aberrant TNFα production ^21^
rno-miR-449a-5p	Bcl2 Prkar2b Tp53 Dffb Fadd Rela	60.2*	1.9	9.8	200.3^†^	1.6^‡^	Induces apoptosis by regulating cyclin D1 and Bcl-2 expression^22^
rno-miR-410-3p	Prkar2b	26.3*	18.8	24.5	141.9^†^	83.3^‡^	Down-regulates the expression of IL-10 through targeting STAT3^23^ and induces apoptosis by targeting high-mobility group box 1 protein (HMGB1)^24^
rno-miR-497-5p	Tradd Il1rap Ripk1 Ikbkb Prkx Cyct Irak2 Prkar2a Dffb Bcl2l1 Map3k14 Nfkb1 Fadd	53.6*	33.7	6.2	107.1^†^	23.1^‡^	Induces apoptosis via the Bcl-2/Bax-Caspase9-Caspase3 pathway^25^
rno-miR-539-5p	Ripk1 Prkx Prkar2b Casp9 Myd88 Ppp3r1 Chuk	15.1*	6.1	11.7	104.3^†^	26.6^‡^	Promotes apoptosis via activation of caspase-3 and suppression of the expression of ERK/AKT and mTOR^26^
rno-miR-29b-3p	Tradd Birc2 Faslg Dffb Tnfrsf1a Casp6 Rela	22.2*	24.7	1.9	90.8^†^	12.6^‡^	Increases the protein level of cleaved caspase‐3 and the protein ratio of Bcl-2 to Bax^27^
rno-miR-34a-5p	Bcl2 Fadd	28.5*	57.3	3.2	60.7^†^	38.0^‡^	Increases caspase-3 and caspase-9 activation and supresses Bcl-2 expression^18^
rno-miR-26b-5p	Ppp3cb	25.2*	2.2	1.4	57.5^†^	1.9^‡^	Increases apoptosis by repression of β‐catenin, and Bcl‐2^30^
rno-miR-98-5p	Il1a Ngf	13.5*	6.2	0.9	32.5^†^	5.2^‡^	Regulates Fas expression and the sensitivity of Fas-mediated apoptosis^31^
rno-miR-152-3p	Chuk	13.7*	10.1	1.0	28.6^†^	10.8^‡^	induces apoptosis by Caspase-3 and Faslg^27^
rno-miR-140-5p	Bcl2l1	13.0*	2.7	1.9	27.8^†^	1.4^‡^	Facilitates the autophagy^32^ and suppresses the TGF-β1 pathway through targeting Smad3^33^
rno-miR-20b-5p	Map3k14 Ppp3r1	9.0	4.1	1.7	21.3^†^	3.4^‡^	Activates the TGF-β signalling pathway^34^
rno-miR-20a-5p	Irak2 Map3k14 Ppp3r1	8.9	7.7	1.5	21.0^†^	6.0^‡^	Mitigates autophagy through suppressing autophagy-related gene 7 (ATG7)^35^ and increases caspase-3 and caspase-9 expression^36^
rno-miR-17-5p	Irak2 Map3k14 Ppp3r1	8.6	4.5	1.5	19.0^†^	3.6^‡^	Suppresses the apoptotic protease activating factor 1 (Apaf-1) expression^37^
rno-miR-323-3p	Dffb Chuk	4.4	2.1	3.7	11.8^†^	10.3^‡^	Suppresses expression of SMAD2 and SMAD3 leading to inactivation of TGF-β signaling^38^ and lowers caspase-3 expression^39^
rno-miR-448-3p	Bcl2 Prkx Prkar2b	2.5	1.1	1.5	9.4^†^	3.7^‡^	Promote apoptosis of nerve cells by downregulating SIRT1^35^

Data from 8 (12-well) plates of the intestinal epithelial cell-6 did not undergo oxygen-glucose deprivation (OGD) (Control-IEC-6 group), 8 (12-well) plates of IEC-6 cell undergo OGD and cultured at 37 °C (OGD-IEC-6+37 °C group), 8 (12-well) plates of IEC-6 cell undergo OGD and cultured at 32 °C (OGD-IEC-6+32 °C group), 8 (12-well) plates of neurons did not undergo OGD and cultured at 37 °C (Control-Neuron group), 8 (12-well) plates of IEC-6 cell undergo OGD then cocultured with neuron at 37 °C (OGD-IEC-6+Neuron+37 °C group), 8 (12-well) plates of IEC-6 cell undergo OGD then cocultured with neuron at 32 °C (OGD-IEC-6+Neuron+32 °C group). The fold increase for each parameter for the control-IEC-6 group is “1”.

OGD, oxygen-glucose deprivation; Caspase, cysteine-containing aspartate-specific protease; ER, endoplasmic reticulum; ADAM10, Bcl-2, B-cell lymphoma 2; STAT3, signal transducer and activator of transcription 3; HMGB1, high-mobility group box 1 protein; ATG5, autophagy-related 5; AREs, adenosine- and uridine-rich elements; Bax, Bcl-2-associated X protein; ERK, extracellular signal-regulated kinase; AKT, protein kinase B; mTOR, mammalian target of rapamycin; ADAM10, A Disintegrin and Metalloproteinase 10; SOX, SRY-related HMG-box; SIRT1, Sirtuin 1; TGF-β, transforming growth factor-beta; IRE1α, inositol-requiring enzyme-1 alpha; TXNIP, thioredoxin-interacting protein; SMAD, small mothers against decapentaplegic; Apaf-1, apoptotic protease activating factor 1.

Only significant and annotated transcripts are indicated.

*OGD-IEC-6-37 °C vs. Control-IEC-6-37 °C.

^†^OGD-IEC-6+Neuron-37 °C vs. Control-IEC-6-37 °C, Control-Neuron-37 °C, or OGD-IEC-6-37°C.

^‡^OGD-IEC-6+Neuron-37 °C vs. OGD-IEC-6+Neuron-32 °C.

## Discussion

As stated in the Introduction section, intestinal I/R injury caused by clamping superior mesenteric artery^4^, or by resuscitation following HS^5^ causes cerebral injury and memory deficits in rats. Our present study provides new evidence to promote that intestinal epithelial cells affected by I/R injury in vitro are able to cause neuronal damage directly. Following OGD, IEC-6 cells can cause cortical neuron death via inducing cellular apoptosis, necroptosis, and pyroptosis events. Additionally, we found that OGD increased intestinal epithelial permeability and decreased the expression of epithelial tight junction-associated proteins. This might allow the spread of many exosomal miRNAs associated with apoptosis, necroptosis, and pyroptosis to injury multiple vital organs in a living organism. Indeed, it has been shown that inflammatory response occurred following I/R injury in rat hearts^6^ and brains^4^ and resuscitation from hemorrhagic shock injury in rat brains^5^.

In our present study, rat intestinal epithelial cells preconditioned with OGD caused accumulation of inflammasome associated with apoptosis, necroptosis, and pyroptosis events in the cocultured medium. The inflammasome is a multiprotein complex comprising caspase-1, the apoptosis-associated speck-like protein containing a C-terminal caspase-activating recruitment domain (ASC), and nucleotide-binding oligomerization domain (NOD)-like receptors (NLRs), such as NLRP-1 or NLRP-3^13^. In the central nervous system, inflammasome-containing microglia, macrophages, and astrocytes are associated with neurological diseases, including stroke^14^. The assembly of an inflammasome activates caspase-1, subsequently cleave GSDMD at an aspartate site and enables the release of the GSDMD N-terminus (GSDMD-N) and then translocates to the plasma membrane to form transmembrane pores. Inflammasome also cleave and activate the proinflammatory cytokines such as IL-1 and IL-18 which are released through GSDMD pore, as well as pyroptotic cell death^15^. Upon activation by miRNAs, a subset of NLR initiates the assembly of the inflammasome, which precesses proinflammatory cytokines and initiates pyroptosis^13^. During pyroptosis, the activation of GSDMD which forms pores in the plasma membrane that cause cellular leakage and lysis, thereby inducing the formation of bubbling and plasma membrane disruption^16^. Since GSDMD-deficient cells resisted the induction of pyroptosis by cytosolic lipopolysaccharide and known canonical inflammasome ligands, GSDMD is required for lipopolysaccharide-induced pyroptosis in HeLa and BMDM cell lines^17^. Additionally, recent studies have demonstrated that caspase‐1 inhibitor Z‐YVAD‐FMK was able to partly reduce GSDMD‐induced pyroptosis after middle cerebral artery occlusion and reperfusion in rats^18^. However, in the present study, although the biochemical features tend to indicate OGD-preconditioned IEC-6 cells can cause cortical neuron death via inducing cellular apoptosis, necroptosis, and pyroptosis events, it still needs to test inhibition of these modes of cell death using major inhibitors in the future studies.

The Rat Inflammatory Response and Autoimmunity miScript microRNA PCR Array kit provide a convenient way to quickly analyze the microRNAs most likely to be relevant to inflammatory and autoimmune disorders. These miRNAs have been carefully selected based on those predicted by bioinformatics algorithms and databases to regulate genes known to be relevant to inflammation^19,20^.

In our present study, the cell culture model of neurons and epithelial cells were separated only by a porous filter. The observed neurotoxicity can be caused by other soluble factors released by damaged IEC-6. The Rat Inflammatory Response and Autoimmunity miScript microRNA PCR Array (from Qiagen) allowed us to assess changes in gene expression of 84 different microRNAs relative to inflammation and immune response pathways. Although we observed that 19 miRNAs related apoptosis, necroptosis, and/or pyroptosis were involved in the pathogenesis of neuronal death, other soluble factors released by damaged cells can not be ruled out in the present results.

**Figure 5:**
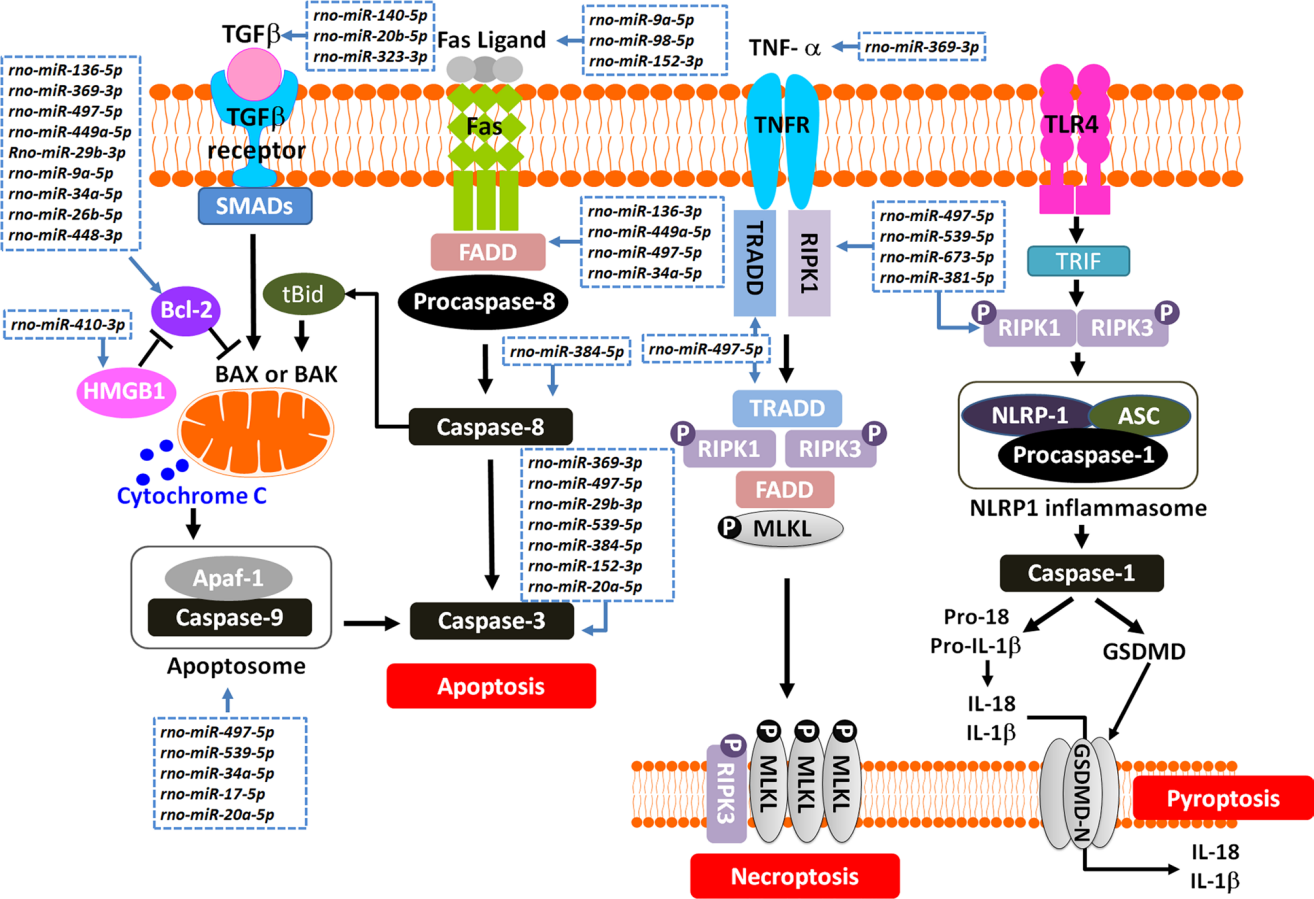
A schematic diagram is linking the upregulated 19 miRNAs in the media of OGD-preconditioned IEC-6 cells and cortical neurons to the events of apoptosis, necroptosis, and pyroptosis that occurred in the cocultured cortical neurons. In particular, pattern recognition receptors (PRRs), such as NOD-like receptors (NLRs) sense conserved microbial signals and host damage signals leading to the coordination of appropriate immune responses. Upon activation, a subset of NLR initiates the assembly of a multimeric protein complex known as the inflammasome, which processes proinflammatory cytokines and mediates pyroptosis^49^. These suggest that paracrine factors released from the injured intestine epithelial cells caused a direct injury to the neurons via the bloodstream in a living situation.

As shown in Table 1, miR-384-5p maintains long-term potentiation of synaptic transmission and enhances stress-induced apoptosis and upregulates cleaved caspase-3 expression^21^. miR-136-5p modulates the inflammatory and immune response and down-regulates Bcl-2^22^. miR-9a-5p regulates the process of autophagy^23^ and increases the levels of the pro-apoptotic regulator^24^. miR-369-3p induces apoptosis^25^ as well as aberrant TNF-α production^26^. miR-449a-5p induces apoptosis by regulating Bcl-2 expression^27^. miR-410-3p downregulates the expression of interleukin-10^28^ and induces apoptosis^29^. miR-497-5p induces apoptosis via the Bcl-2/Bax-caspase-9-caspase-3 pathway^30^. miR-539-5p promotes apoptosis via caspase-3 activation and the expression of ERK/AKT and mTOR^31^. miR-29b-3p increases the cleaved caspase-3 and the ratio of Bcl-2 to Bax^32^. miR-34a-5p increases caspase-3 and caspase-9 activation, suppress Bcl-2 expression^33^ and increases free-radicals accumulation^34^.miR-26b-5p increases apoptosis by repression of β-catenin and Bcl-2^35^. miRNA-98-5p regulates Fas expression and apoptosis^36^. miR-152-3p induces apoptosis by caspase-3 and Faslg^32^. miR-140-5p facilitates the autophagy^37^ and suppresses the TGF-β1 pathway^38^. miR-20b-5p activates TGF-β signaling pathway^39^. miR-20a-5p mitigates autophagy^40^ and increases caspase-3 and caspase-9 expression^41^. miR-17-5p suppresses the apoptotic protease activating factor (Apaf-1) expression^42^. miR-323-3p suppresses SMAD2/3, inactivates TGF-β signaling^43^and lowers caspase-3 expression^44^. miR-448-3p promotes apoptosis by down-regulating SIRT1^40^. It can be derived from the above-mentioned observation and KEGG pathway analysis of their target gene, these 19 up-regulated miRNAs profiles are involved in the pathogenesis of apoptosis, necroptosis, and pyroptosis that occurred in the cortical neurons cocultured with OGD-preconditioned IEC-6 cells (Fig. 5). Our data suggest that the injured intestine epithelial cells can damage neurons directly via the paracrine factors. In addition, Figs. 4, 5, and Table 1 showed that all the 19 exosomal miRNAs upregulated in cortical neurons co-cultured with OGD-IEC-6 cells were significantly attenuated by therapeutic hypothermia (OGD-IEC-6+Neuron+32 °C group).

Hemorrhagic shock (HS) is a predictor of poor outcomes in trauma patients. Early hypotension with hemorrhage may cause multiple organ failure and secondary infection. In the USA and Europe, blood and fluid volume replacement are the current resuscitation strategies for the management of HS^45^. However, these transfusions may themselves cause the development of multiple organ failure and increased mortality via intestinal I/R injury^45^. Therapeutic hypothermia (32–34 °C) improves the outcome of HS by reducing the resuscitation fluid volumes required to maintain blood pressure, the expression of reactive oxygen species, as well as microvascular permeability^46^. Hypothermia may also preserve adenosine triphosphate and glycogen stores^47^ and extracellular signal-regulated kinases in ischemic tissue^48^, which resulted in reduced inflammatory response and decreased cell death^6^. Our present data further suggest that therapeutic hypothermia improves outcomes following HS by reducing the inflammatory response following I/R injury in the intestine.

Our previous report has demonstrated that a unilateral common carotid artery occlusion (UCCAO) causes slight cerebral ischemia (cerebral blood flow [CBF] from 100% downed to 70%), insignificant hypotension (MABP 85 mmHg), systemic inflammation, multiple organs injuries, or neurological injury^5^. An HS caused moderate cerebral ischemia (52% of the original CBF levels), moderate hypotension (MABP downed to 30 mmHg), systemic inflammation, and peripheral organ injuries. However, combined a UCCAO and an HS caused severe cerebral ischemia (18% of the original CBF levels), severe hypotension (MABP downed to 20 mmHg), systemic inflammation, peripheral organ damage, and neurological injury, which can be attenuated by therapeutic hypothermia. Our present results further showed that therapeutic hypothermia did not affect the hyperpermeability of the OGD-preconditioned IEC-6 layer in vitro but did significantly ameliorate the overproduction of 19 exosomal miRNAs related apoptosis, necroptosis, and pyroptosis events and cortical neuron death caused by OGD-preconditioned IEC-6 cells.

**Figure 6:**
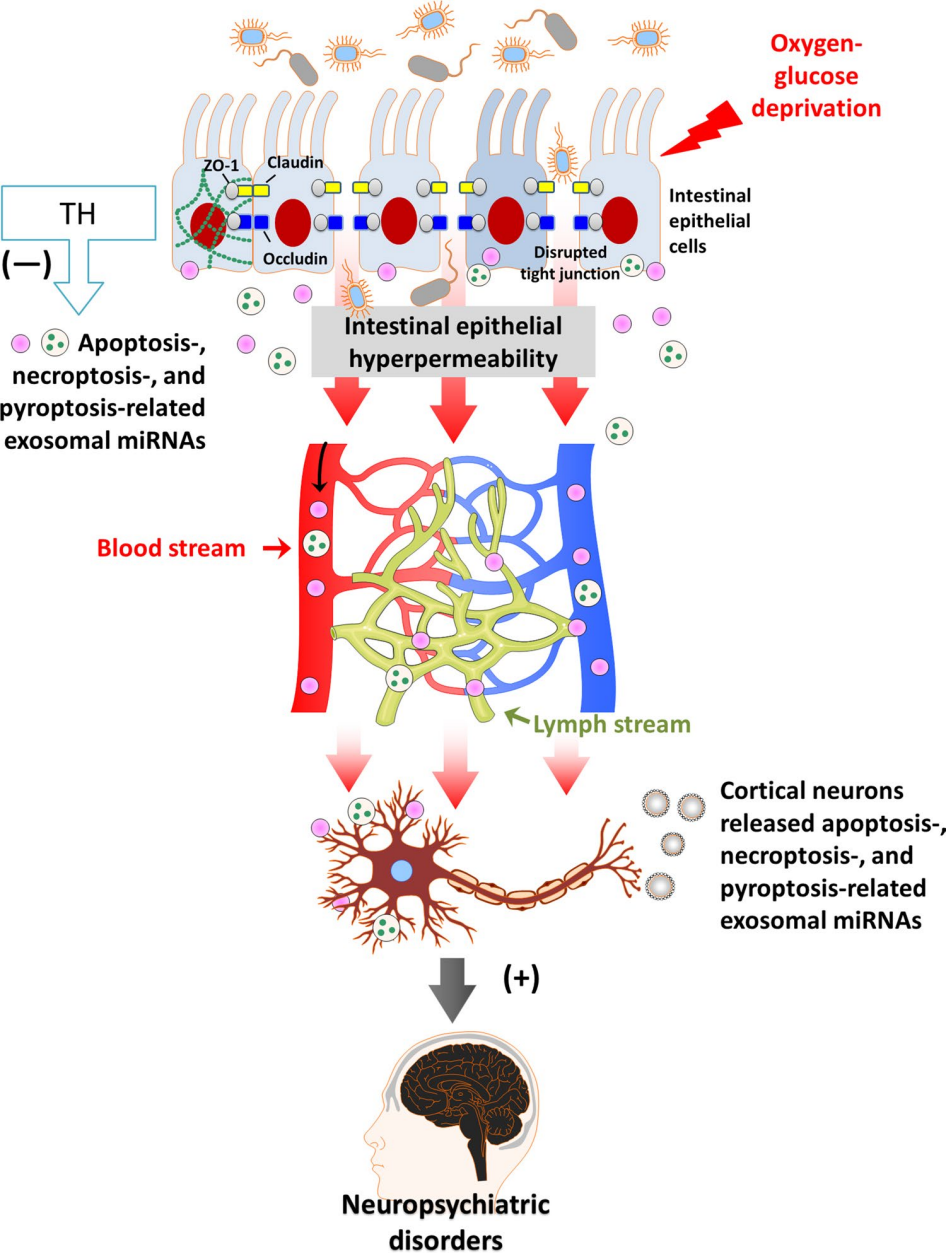
Overview of the “gut-brain” theory based on the present results. In summary, our data show that OGD causes intestinal epithelial hyperpermeability and the release of many exosomal pyroptosis-, necroptosis, and apoptosis-related miRNAs. Then, these proteins pass through the hyperpermeated intestinal epithelium translocated into the blood and lymph routes and induced cortical neuronal apoptosis, necroptosis, and pyroptosis, which may result in the occurrence of neuropsychiatric disorders. Although therapeutic hypothermia (TH: 32 °C) did not affect the intestinal epithelial hyperpermeability, they did ameliorate the cortical neuronal apoptosis, necroptosis, and pyroptosis caused by OGD-preconditioned intestinal epithelial cells. ( +) exacerbation; (−) amelioration.

Based on previous^1^ and present results, in living organisms, OGD may cause intestinal epithelial hyperpermeability and the release of many apoptosis, necroptosis, and pyroptosis-related exosomal miRNAs. These released mediators may be translocated into the blood and lymph routes to induce cortical neuronal apoptosis, necroptosis, and pyroptosis, which result in the occurrence of neuropsychiatric disorders. Therapeutic hypothermia did not affect the gut barrier but did attenuate the cortical neuronal death caused by OGD-preconditioned epithelial cells (Fig. 6). TH might attenuate OGD-IEC-6 induced cortical neuronal death by reducing the inflammatory response following I/R injury in the intestine.

The protective effects of TH are a combined effect on neurons and intestinal epithelial cells. In future studies, we aim to ascertain whether the brain injury caused by the superior mesentery artery ligation or by resuscitation following hemorrhagic shock can be affected by selective brain cooling in vivo.

## Conclusion

Our results show that OGD causes intestine epithelial hyperpermeability in IEC-6 cell layer, overproduction of neuronal apoptosis-related (e.g., increased expression of caspase-3), necroptosis-related (increased expression of RIPK1, RIPK3, and MLKL), pyroptosis-related (increased expression of caspase-1, GSDMD, GSDMD-N, ASC and NLRP-1 inflammasome complex) exosomal miRNAs and synaptotoxicity. Although therapeutic hypothermia did not affect the hyperpermeability of the OGD-preconditioned IEC-6 layer in vitro, it did significantly ameliorate the overproduction of 19 exosomal miRNAs and cortical neuronal death caused by OGD-preconditioned IEC-6 cells.

## Supplementary information

Supplementary file1

## Data Availability

The datasets used and/or analyzed during the current study are available from the corresponding author on reasonable request.

## Abbreviations

ASC: Apoptosis-associated speck-like protein containing a C-terminal caspase recruitment domain
Caspase-1: Cysteine-aspartic protease-1
Caspase-3: Cysteine-aspartic protease-3
Caspase-8: Cysteine-aspartic protease-8
DAPI: 4′,6-Diamidino-2-phenylindole dihydrochloride
ELISA: Enzyme-linked immunosorbent assay
GSDMD: GasderminD
GSDMD-N: GSDMD N-terminus
HS: Hemorrhagic shock
I/R: Ischemia/reperfusion
IACUC: Institutional animal care and use committee
IEC-6: Intestinal epithelial cell-6
IL-1β: Interleukin-1 beta
IL-18: Interleukin-18
IL-6: Interleukin-6
MAP2: Microtubule-associated protein-2
MTT: 3-(4,5-Dimethylthiazol-2-yl)-2,5-diphenyltetrazolium bromide
NC: Normal control
NLRP-1: Nucleotide-binding oligomerization domain (NOD)-like receptor protein 1
NLRP-3: Nucleotide-binding oligomerization domain (NOD)-like receptor protein 3
OGD: Oxygen–glucose deprivation
PBS: Phosphate-buffered saline
RIPK1: Receptor-interacting protein kinase-1
RIPK3: Receptor-interacting protein kinase-3
pMLKL: Phosphorylated-mixed lineage kinase domain-like pseudokinase
TEER: Transepithelial electrical resistance
TNF-α: Tumour necrosis factor alpha
TUNEL: Terminal deoxynucleotidyl transferase dUTP nick end labelling
ZO-1: Zonula occludens 1
