# Supplementary material for: Ischemia/reperfusion injured intestinal epithelial cells cause cortical neuron death by releasing exosomal microRNAs associated with apoptosis, necroptosis, and pyroptosis
Source: Sci Rep. 2020 Sep 1;10:14409. doi: 10.1038/s41598-020-71310-5 (PMC7462997; doi:10.1038/s41598-020-71310-5)
Supplement: Supplementary file 1 — Supplementary file1 [file 41598_2020_71310_MOESM1_ESM.docx]

**Supplemental Data**

**Ischemia/reperfusion injured intestinal epithelial cells cause cortical neuron death by releasing exosomal microRNAs associated with apoptosis, necroptosis, and pyroptosis**

Chien-Chin Hsu, Chien-Cheng Huang, Lan-Hsiang Chien, Mao-Tsun Lin, Ching-Ping Chang, Hung-Jung Lin, Chung-Ching Chio

***Correspondence to:**

Ching-Ping Chang, e-mail: [jessica.cpchang@gmail.com](mailto:jessica.cpchang@gmail.com) or [a50831@mail.chimei.org.tw](mailto:a50831@mail.chimei.org.tw)

Hung-Jung Lin, e-mail: [790001@mail.chimei.org.tw](mailto:790001@mail.chimei.org.tw)

Chung-Ching Chio, e-mail: [chiocc@ms28.hinet.net](mailto:chiocc@ms28.hinet.net)

**This word file includes:**

**Tables S1-S2**

**Figure S1-S3**

| **Supplementary Table S1. Antibodies and kits used in immunofluorescence staining (IF), Western blotting (WB), and ELISA.** | | | | |
| --- | --- | --- | --- | --- |
| **Antibody** | **Company** | **Titre** | **Catalogue #** | **Purpose** |
| MAP2 | Santa Cruz | 1:100 | sc-74421 | IF |
| Synaptophysin | ThermoFisher | 1:100 | MA5-14532 | IF |
| Caspase-1 | Abcam, | 1:1000 | ab179515 | WB |
| Caspase-3 | Cell Signaling | 1:2000 | 9665 | WB |
| Caspase-8 | Cell Signaling | 1:2000 | 4790 | WB |
| NLRP-1 | Cell Signaling | 1:1000 | 4990 | WB |
| NLRP-3 | Abcam | 1:1000 | ab210491 | WB |
| ASC | Santa Cruz | 1:1000 | sc-514414 | WB |
| IL-1β | Abcam | 1:2500 | ab9787 | WB |
| CD9 | St John’s | 1:1000 | STJ92147 | WB |
| RIPK1 | Cell Signaling | 1:2000 | 3493 | WB |
| RIPK3 | Cell Signaling | 1:2000 | 15828 | WB |
| p-MLKL (Ser358) | Cell Signaling | 1:2000 | 91689 | WB |
| GSDMD | Cell Signaling | 1:2000 | 93709 | WB |
| ZO-1 | Abcam | 1:7500 | ab96587 | WB |
| Claudin-1 | Abcam | 1:750 | ab15098 | WB |
| β-actin | Santa Cruz | 1:5000~10000 | sc-47778 | WB |
| Rat IL-1β ELISA kit | R & D Systems | according to the manufacturer protocol | DY501 | ELISA |
| Rat IL-6 ELISA kit | BD Biosciences | according to the manufacturer protocol | 550319 | ELISA |
| Rat TNF-α ELISA kit | BD Biosciences | according to the manufacturer protocol | 558535 | ELISA |
| Rat IL-18 ELISA kit | Abcam | according to the manufacturer protocol | ab213909 | ELISA |
| 4’6-diamidino-2-phenylindole (DAPI) | Thermo Fisher | 1: 5000~25000 | 62247 | IF |
| Secondary antibody Alexa Fluor 488 (rabbit IgG) | Invitrogen | 1:400 | A21441 | IF |
| Secondary antibody Alexa Fluor 488 (rabbit IgG) | Invitrogen | 1:400 | A11008 | IF |
| Secondary antibody Alexa Fluor 568 (mouse IgG) | Invitrogen | 1:400 | A11004 | IF |
| Anti-rabbit IgG, HRP-linked antibody | Cell Signaling | 1:2000~20000 | 7074 | WB |
| Anti-mouse IgG, HRP-linked antibody | Cell Signaling | 1:2000~5000 | 7076S | WB |
| Anti-mouse IgG, HRP-linked antibody | Santa Cruz | 1:2000~5000 | Sc-4778 | WB |
| ApoAlertTM DNA Fragmentation Assay Kit | Clontech Laboratories | according to the manufacturer protocol | 630107 | IF |
| RNase-Free DNase Set | Qiagen | according to the manufacturer protocol | 79254 | IF |
| miRNeasy Mini kits | Qiagen | according to the manufacturer protocol | 217084 | Exosome purifictaion |
| Rat Inflammatory Response & Autoimmunity miScript miRNA PCR Array platform | Qiagen | according to the manufacturer protocol | 331221 | qRT-PCR |

**Abbreviations:** NeuN, neuronal nuclei; TNF-α, tumour necrosis factor-alpha; MAP2, Microtubule-associated protein 2; Caspase, cysteine-containing aspartate-specific protease; NLRP, nucleotide-binding oligomerization domain (NOD)-like receptor protein; ASC, apoptosis-associated speck-like protein containing a C-terminal caspase recruitment domain; IL, interleukin; RIPK, receptor-interacting serine/threonine protein kinase; pMLKL, phospho-mixed lineage kinase domain-like protein; DAPI, 4’,6-diamidino-2-phenylindole dihydrochloride; HRP, horseradish peroxidase.

**Supplementary Table S2**

Fold changes of 84 exosomal miRNAs expression in the media of Control-IEC-6 cells, OGD preconditioned-IEC-6 at 37 ^o^C (OGD-IEC-6+37 ^o^C), OGD preconditioned-IEC-6 at 32 ^o^C (OGD-IEC-6+32 ^o^C),control cortical neurons at 37 ^o^C (Control Neuron+37 ^o^C), cortical neurons cocultured with OGD-preconditioned IEC-6 cells at 37 ^o^C (OGD-IEC-6+Neuron+37 ^o^C) and cortical neurons cocultured with OGD-preconditioned IEC-6 cells at 32 ^o^C (OGD-IEC-6+Neuron+32 ^o^C).

| **Commercialized**  **Kit**  **Position** | **Mature ID** | **Fold Change [ comparing to (Control - IEC-6) group]** | | | | |
| --- | --- | --- | --- | --- | --- | --- |
|  |  | **OGD - IEC-6+**  **37 ^o^C** | **OGD-**  **IEC-6-+**  **32 ^o^C** | **Control – Neuron+**  **37 ^o^C** | **OGD-**  **IEC-6 + Neuron+**  **37 ^o^C** | **OGD - IEC-6+ Neuron+**  **32 ^o^C** |
| A01 | rno-let-7a-5p | 3.3106 | 1.0177 | 0.272 | 4.7477 | 0.2177 |
| A02 | rno-let-7b-5p | 12.4494 | 3.7445 | 0.7124 | 15.2767 | 2.9587 |
| A03 | rno-let-7c-5p | 18.4865 | 4.8715 | 0.6336 | 25.5323 | 4.147 |
| A04 | rno-let-7d-5p | 2.9818 | 3.6567 | 0.2738 | 4.7785 | 1.6953 |
| A05 | rno-let-7e-5p | 8.4347 | 2.0365 | 0.4707 | 13.0002 | 0.9756 |
| A06 | rno-let-7f-5p | 4.3724 | 1.7059 | 0.2889 | 9.9158 | 0.648 |
| A07 | rno-let-7i-5p | 13.3262 | 4.4806 | 0.8151 | 20.9558 | 3.2185 |
| A08 | rno-miR-101a-3p | 17.0559 | 6.6979 | 3.2007 | 31.1563 | 5.6495 |
| A09 | rno-miR-101b-3p | 33.4131 | 15.3556 | 3.965 | 53.1006 | 11.8096 |
| A10 | rno-miR-106b-5p | 10.7115 | 10.0767 | 1.9024 | 16.3494 | 15.4966 |
| A11 | rno-miR-125a-5p | 4.8116 | 1.7435 | 0.2206 | 2.7581 | 2.8098 |
| A12 | rno-miR-125b-5p | 30.248 | 13.8118 | 2.6937 | 32.6842 | 22.3839 |
| B01 | rno-miR-128-3p | 5.6873 | 4.0789 | 0.9257 | 2.3602 | 17.6614 |
| B02 | **rno-miR-136-5p** | 119.9362 | 67.6877 | 18.1147 | 629.5488 | 87.9864 |
| B03 | **rno-miR-140-5p** | 12.99 | 2.7159 | 1.8988 | 27.7533 | 1.3902 |
| B04 | rno-miR-141-3p | 16.3417 | 5.4722 | 0.399 | 13.9032 | 5.0156 |
| B05 | rno-miR-142-3p | 5.3438 | 6.2505 | 0.637 | 3.9857 | 4.6456 |
| B06 | rno-miR-144-3p | 16.1933 | 0.3927 | 1.5696 | 29.9351 | 1.2441 |
| B07 | rno-miR-145-5p | 4.3105 | 19.6636 | 0.628 | 6.6263 | 84.2671 |
| B08 | rno-miR-148b-3p | 9.7295 | 3.6743 | 0.8337 | 15.9503 | 4.1162 |
| B09 | **rno-miR-152-3p** | 13.7442 | 10.1169 | 0.9952 | 28.6469 | 10.7894 |
| B10 | rno-miR-15b-5p | 11.5545 | 5.6192 | 0.9972 | 16.1677 | 4.0874 |
| B11 | rno-miR-16-5p | 32.5042 | 7.8559 | 2.4721 | 59.9074 | 7.7239 |
| B12 | **rno-miR-17-5p** | 8.6236 | 4.5406 | 1.5496 | 19.0255 | 3.5975 |
| C01 | rno-miR-181a-5p | 39.7472 | 14.4885 | 1.5226 | 24.9199 | 42.7075 |
| C02 | rno-miR-181b-5p | 25.4382 | 6.0846 | 1.9812 | 24.2511 | 11.6377 |
| C03 | rno-miR-181c-5p | 64.7636 | 26.5388 | 2.2499 | 58.5825 | 80.9487 |
| C04 | rno-miR-181d-5p | 16.8135 | 4.3185 | 1.67 | 15.009 | 5.7551 |
| C05 | rno-miR-182 | 9.4819 | 2.8768 | 0.2879 | 7.2259 | 4.3783 |
| C06 | rno-miR-183-5p | 11.418 | 4.3104 | 0.8257 | 13.1834 | 3.3421 |
| C07 | rno-miR-186-5p | 8.6801 | 6.1635 | 0.5032 | 7.1705 | 8.7094 |
| C08 | rno-miR-195-5p | 28.6358 | 8.1523 | 1.5588 | 51.9524 | 6.8373 |
| C09 | rno-miR-19a-3p | 62.1704 | 38.9115 | 7.9896 | 122.2847 | 58.1066 |
| C10 | rno-miR-19b-3p | 66.6037 | 47.3757 | 7.3719 | 123.0728 | 73.5311 |
| C11 | rno-miR-200a-3p | 33.5912 | 16.8064 | 0.341 | 36.5451 | 10.4817 |
| C12 | rno-miR-200c-3p | 16.7625 | 6.6439 | 0.1433 | 22.5713 | 3.6838 |
| D01 | rno-miR-203a-3p | 1.0787 | 0.1939 | 0.1929 | 1.9239 | 0.5022 |
| D02 | rno-miR-205 | 19.9272 | 0.0665 | 2.1189 | 29.1346 | 0.1415 |
| D03 | **rno-miR-20a-5p** | 8.8933 | 7.7421 | 1.4922 | 20.9573 | 6.0072 |
| D04 | **rno-miR-20b-5p** | 9.018 | 4.1201 | 1.6857 | 21.3437 | 3.3656 |
| D05 | rno-miR-21-5p | 24.5067 | 11.6921 | 1.318 | 46.3043 | 5.54 |
| D06 | rno-miR-221-3p | 11.2306 | 10.3236 | 0.8083 | 19.9229 | 17.2032 |
| D07 | rno-miR-222-3p | 3.881 | 2.9789 | 0.3769 | 3.986 | 4.4875 |
| D08 | rno-miR-23a-3p | 13.8522 | 3.904 | 0.2797 | 12.5877 | 3.1592 |
| D09 | rno-miR-23b-3p | 13.1471 | 6.7498 | 0.1444 | 9.9532 | 2.7927 |
| D10 | rno-miR-26a-5p | 40.4484 | 20.967 | 2.6498 | 73.6052 | 28.934 |
| D11 | **rno-miR-26b-5p** | 25.2444 | 2.1798 | 1.3953 | 57.5276 | 1.8682 |
| D12 | rno-miR-27a-3p | 7.5226 | 1.2045 | 1.0773 | 10.3381 | 0.7356 |
| E01 | rno-miR-27b-3p | 5.9046 | 3.7937 | 0.5135 | 7.5514 | 3.287 |
| E02 | rno-miR-291a-3p | 0.5936 | 1.2852 | 1.5286 | 2.4155 | 3.8882 |
| E03 | rno-miR-29a-3p | 5.2692 | 1.7503 | 0.7012 | 6.8444 | 2.1014 |
| E04 | **rno-miR-29b-3p** | 22.1951 | 24.7344 | 1.8669 | 90.0799 | 12.6343 |
| E05 | rno-miR-29c-3p | 5.2456 | 1.6819 | 0.7386 | 6.7852 | 1.8737 |
| E06 | rno-miR-30a-5p | 32.5542 | 12.3047 | 2.5018 | 35.4961 | 31.5855 |
| E07 | rno-miR-30b-5p | 8.3864 | 1.3486 | 0.3829 | 1.4887 | 0.6976 |
| E08 | rno-miR-30c-5p | 15.5688 | 4.6621 | 0.2337 | 5.7811 | 3.9001 |
| E09 | rno-miR-30d-5p | 21.7653 | 7.3422 | 1.2026 | 22.0799 | 23.7692 |
| E10 | rno-miR-30e-5p | 32.854 | 9.166 | 2.8655 | 42.3842 | 12.6862 |
| E11 | rno-miR-320-3p | 0.6365 | 0.2447 | 0.4548 | 0.9342 | 1.1821 |
| E12 | rno-miR-322-5p | 14.8237 | 9.9552 | 2.465 | 25.7499 | 3.875 |
| F01 | **rno-miR-323-3p** | 4.4348 | 2.105 | 3.7103 | 11.7779 | 10.3329 |
| F02 | rno-miR-325-3p | 44.6909 | 39.4501 | 16.0607 | 67.8313 | 65.263 |
| F03 | rno-miR-327 | 0.4367 | 0.4708 | 0.9403 | 1.3126 | 1.8464 |
| F04 | **rno-miR-34a-5p** | 28.5348 | 57.3754 | 3.1845 | 60.7211 | 38.0433 |
| F05 | rno-miR-34c-5p | 104.2298 | 194.0678 | 3.8767 | 161.4576 | 50.1015 |
| F06 | rno-miR-351-5p | 7.8968 | 7.5075 | 0.1942 | 3.642 | 9.4981 |
| F07 | **rno-miR-369-3p** | 35.8128 | 5.66 | 40.5503 | 248.038 | 25.3188 |
| F08 | rno-miR-374-5p | 18.862 | 8.4129 | 0.8068 | 29.801 | 5.7175 |
| F09 | rno-miR-381-3p | 0.9891 | 0.7538 | 0.6113 | 2.4984 | 1.5937 |
| F10 | **rno-miR-384-5p** | 164.6163 | 125.3726 | 35.5988 | 782.9853 | 267.7238 |
| F11 | **rno-miR-410-3p** | 26.3371 | 18.8421 | 24.5188 | 141.895 | 83.2953 |
| F12 | rno-miR-429 | 10.4544 | 3.9509 | 0.1254 | 7.4699 | 4.3299 |
| G01 | **rno-miR-448-3p** | 2.45 | 1.138 | 1.4834 | 9.3789 | 3.7246 |
| G02 | **rno-miR-449a-5p** | 60.2022 | 1.8803 | 9.7576 | 200.2836 | 1.5975 |
| G03 | rno-miR-495 | 27.5524 | 20.4335 | 30.8244 | 19.9827 | 86.7688 |
| G04 | **rno-miR-497-5p** | 53.6469 | 33.7098 | 6.1607 | 107.0616 | 23.0525 |
| G05 | **rno-miR-539-5p** | 15.0618 | 6.0486 | 11.7374 | 104.3417 | 26.599 |
| G06 | rno-miR-664-3p | 3.0318 | 1.4742 | 0.0261 | 1.4808 | 2.6723 |
| G07 | rno-miR-673-5p | 1.155 | 1.3609 | 0.5989 | 1.8808 | 3.6153 |
| G08 | rno-miR-743b-3p | 2.6008 | 1.077 | 0.2887 | 7.3049 | 1.4224 |
| G09 | rno-miR-878 | 2.3512 | 0.7384 | 1.3753 | 4.6494 | 4.6812 |
| G10 | **rno-miR-9a-5p** | 67.3893 | 100.2481 | 49.5775 | 403.8749 | 239.7014 |
| G11 | rno-miR-93-5p | 7.0962 | 3.0536 | 1.1816 | 14.1663 | 3.0013 |
| G12 | **rno-miR-98-5p** | 13.4987 | 6.1946 | 0.8505 | 32.45 | 5.1884 |
| H01 | cel-miR-39-3p | 0.0527 | 0.0292 | 0.0556 | 0.0949 | 0.1355 |
| H02 | cel-miR-39-3p | 0.0282 | 0.0144 | 0.0299 | 0.0476 | 0.0739 |
| H03 | SNORD61 | 7.5853 | 2.967 | 1.9101 | 14.1177 | 4.9948 |
| H04 | SNORD68 | 1.0317 | 1.1731 | 0.9831 | 0.4772 | 0.9636 |
| H05 | SNORD72 | 13.4858 | 180.4438 | 0.2964 | 16.4969 | 18.4256 |
| H06 | SNORD95 | 0.9692 | 0.8524 | 1.0172 | 2.0955 | 1.0378 |
| H07 | SNORD96A | 6.0382 | 12.8033 | 0.407 | 5.4311 | 17.3474 |
| H08 | RNU6-6P | 4.4506 | 23.5503 | 0.7667 | 4.8926 | 7.94 |
| H09 | miRTC | 0.0454 | 0.1277 | 0.0461 | 0.1114 | 0.4662 |
| H10 | miRTC | 0.0417 | 0.1184 | 0.0444 | 0.1064 | 0.3735 |
| H11 | PPC | 0.1227 | 0.1603 | 0.0708 | 0.3104 | 0.2392 |
| H12 | PPC | 0.1081 | 0.1478 | 0.0652 | 0.2804 | 0.1839 |


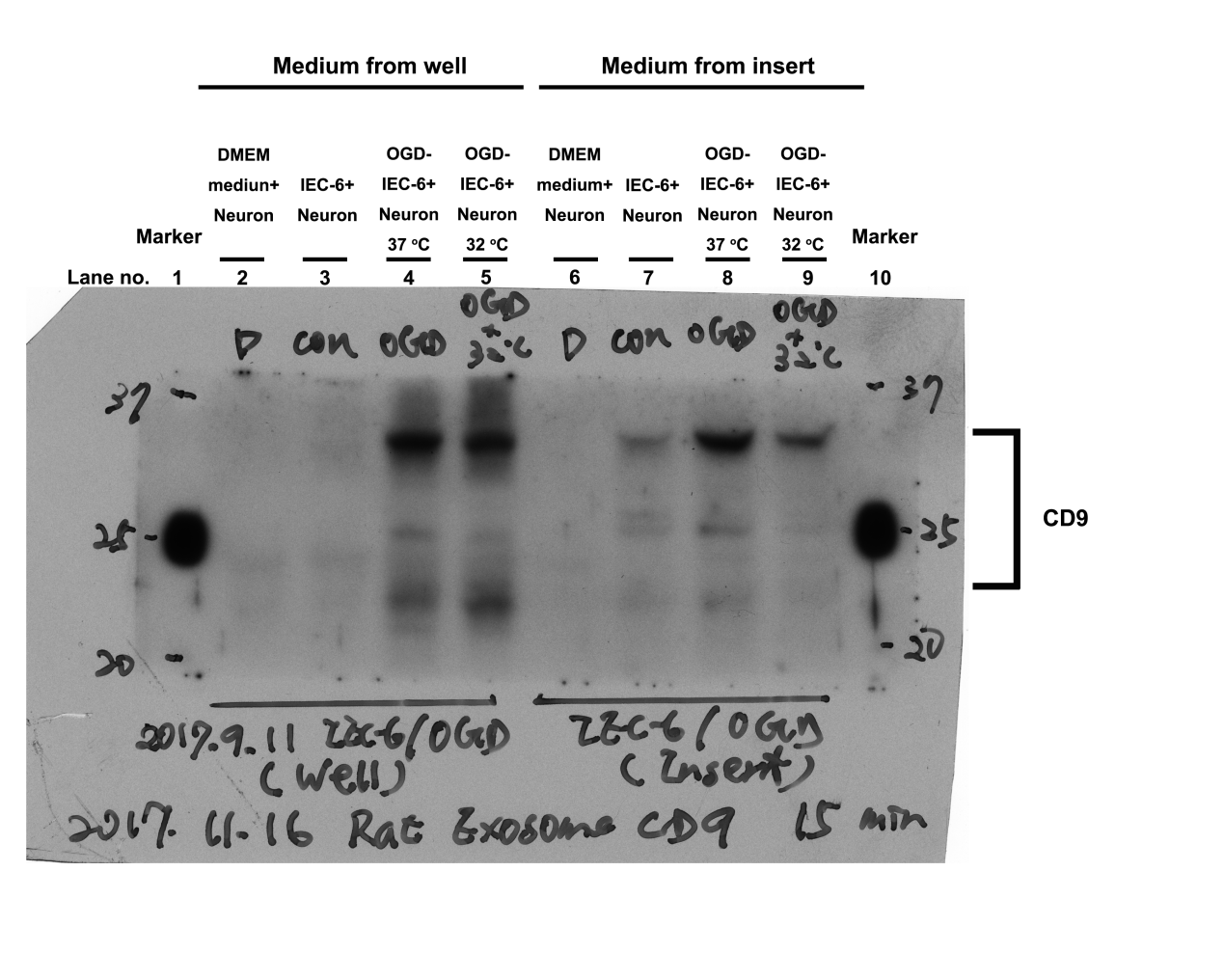


**Supplementary Figure S1**: The efficacy of exosome extraction in all samples was confirmed using Western blot for CD9 (22~27 kDa). Exosomes were isolated separately from the culture medium of the well and insert. Lane 1 and 10 are molecular weight markers. Lane 2 to 5 are the medium from the well of DMEM medium with neuron coculture, IEC-6 with neuron coculture, OGD-IEC-6 with neuron coculture under 37 ^o^C, and OGD-IEC-6 with neuron co-culture under 32 ^o^C group. Lane 6 to 9 are the medium from the insert of DMEM medium with neuron coculture, IEC-6 with neuron coculture, OGD-IEC-6 with neuron coculture under 37 ^o^C, and OGD-IEC-6 with neuron co-culture under 32 ^o^C group.


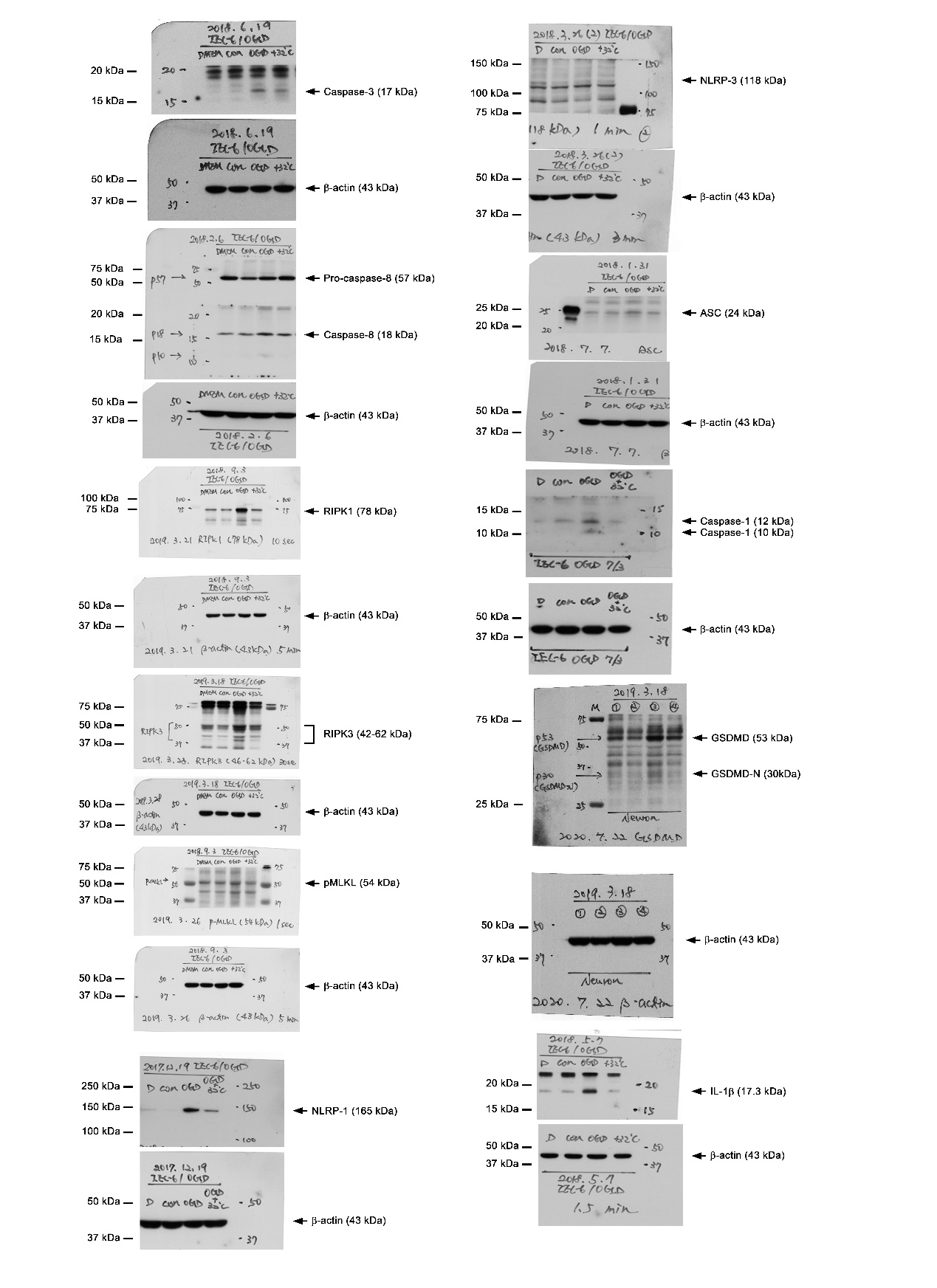


**Supplementary Figure S2:** Original Western blots for Figure 3 (A). Labels on the right of each x-film scan indicate the antibody used for detection. Dashed red boxes show the regions that were cropped for the figures.

**

**

**Supplemental Figure S3:** Hypothermia therapy did not affect the tight junction-associated proteins (ZO-1 and claudin-1) expression of the IEC-6 cells. Lane ➀ indicate IEC-6 under standard culture condition at 37 ^o^C for 36 hours. Lane ➁ indicates the IEC-6 with OGD cultured for 24 hours at 37 ^o^C and then returned to standard culture condition at 37 ^o^C for 12 hours. Lane ➂ indicates the IEC-6 with OGD cultured 24 hours at 37 ^o^C and then at 32 ^o^C. Labels on the right of each x-film scan indicate the antibody used for detection. Labels on the left of each x-film scan indicate the markers used for the evaluation of molecular weight. Dashed red boxes show the regions of (A) ZO-1 and its internal control β-actin protein expression and (B) claudin-1and its internal control β-actin protein expression.
